# System-level investigation of anti-obesity effects and the potential pathways of *Cordyceps militaris* in ovariectomized rats

**DOI:** 10.1186/s12906-022-03608-y

**Published:** 2022-05-12

**Authors:** Dongyeop Jang, Eunjoo Lee, Sullim Lee, Yongsam Kwon, Ki Sung Kang, Chang-Eop Kim, Daeyoung Kim

**Affiliations:** 1 Department of Physiology, College of Korean Medicine, Gachon University, Seongnam, 13120 Korea; 2 Department of Life Science, College of Bio-Nano Technology, Gachon University, Seongnam, 13120 Korea; 3 Dong-A Pharmaceutical Co., LTD, Yongin, 17073 Korea; 4 Department of Preventive Medicine, College of Korean Medicine, Gachon University, Seongnam, 13120 Korea

**Keywords:** *Cordyceps militaris*, Menopause, Obesity, Estrogen receptor, Mitogen-activated protein kinase, Network pharmacology

## Abstract

**Background:**

*Cordyceps* species have been used as tonics to enhance energy, stamina, and libido in traditional Asian medicine for more than 1600 years, indicating their potential for improving reproductive hormone disorders and energy metabolic diseases. Among *Cordyceps*, *Cordyceps militaris* has been reported to prevent metabolic syndromes including obesity and benefit the reproductive hormone system, suggesting that *Cordyceps militaris* can also regulate obesity induced by the menopause. We investigated the effectiveness of *Cordyceps militaris* extraction (CME) on menopausal obesity and its mechanisms.

**Methods:**

We applied an approach combining in vivo, in vitro, and in silico methods. Ovariectomized rats were administrated CME, and their body weight, area of adipocytes, liver and uterus weight, and lipid levels were measured. Next, after the exposure of MCF-7 human breast cancer cells to CME, cell proliferation and the phosphorylation of estrogen receptor and mitogen-activated protein kinases (MAPK) were measured. Finally, network pharmacological methods were applied to predict the anti-obesity mechanisms of CME.

**Results:**

CME prevented overweight, fat accumulation, liver hypertrophy, and lowered triglyceride levels, some of which were improved in a dose-dependent manner. In MCF-7 cell lines, CME showed not only estrogen receptor agonistic activity through an increase in cell proliferation and the phosphorylation of estrogen receptors, but also phosphorylation of extracellular-signal-regulated kinase and p38. In the network pharmacological analysis, bioactive compounds of CME such as cordycepin, adenine, and guanosine were predicted to interact with non-overlapping genes. The targeted genes were related to the insulin signaling pathway, insulin resistance, the MARK signaling pathway, the PI3K–Akt signaling pathway, and the estrogen signaling pathway.

**Conclusions:**

These results suggest that CME has anti-obesity effects in menopause and estrogenic agonistic activity. Compounds in CME have the potential to regulate obesity-related and menopause-related pathways. This study will contribute to developing the understanding of anti-obesity effects and mechanisms of *Cordyceps militaris*.

**Supplementary Information:**

The online version contains supplementary material available at 10.1186/s12906-022-03608-y.

## Background

Menopause leads to various physical and mental symptoms, such as daily hot flashes, a lack of energy, back pain, breast pain, anxiety, and poor memory [1]. Menopause is also considered to be related to obesity [1], because estrogen plays an important role in the energy balance and metabolism of adipose tissue and other organs [2]. Indeed, the prevalence rate of overweight in women over the age of 45 is higher than that in men, whereas the trend is reversed among young adults, suggesting that obesity coincides with menopause in women [3]. There is a report showing the interaction between reduced estrogen secretion and obesity in menopausal women, implying that estrogen deficiency in menopausal women contributes to obesity [4]. Obesity is believed to increase the risk of type 2 diabetes mellitus, cardiovascular diseases, and osteoarthritis [5]. Thus, the World Health Organization has classified obesity as a preventable cause of death [6]. However, the prevalence of obesity has increased to the point that nearly one-third of the world’s population was classified as overweight or obese in 2019 [3], and the prevalence of adult obesity and severe obesity will continue to increase [7, 8].

Hormone replacement therapy (HRT), also known as menopausal hormone therapy or postmenopausal hormone therapy, is used to treat various symptoms involved in menopause [9], and it has been reported that HRT can alleviate obesity and its related symptoms [10–12]. Many women, however, avoid HRT due to its adverse effects and choose non-hormonal therapies [13, 14]. As an alternative, natural products have been studied as effective and safe drugs for relieving postmenopausal diseases and symptoms [15–18]. Systematic reviews have shown that some natural products significantly inhibit weight gain in animals and humans without severe adverse events or mortality [19, 20]. In addition, it has been reported that herbal extracts proliferate estrogen receptor (ER)-positive MCF-7 cells, and increase the mRNA expression of estrogen-related genes [21–23], suggesting that herbal compounds can improve estrogen-deficiency-related menopause symptoms through their estrogenic effects.

The fungal genus *Cordyceps* is parasitic on Lepidopteron insects, and the combination of the fungi and dead insect has been used as a drug in traditional Asian medicine. *Cordyceps* species are traditionally called ‘winter worm summer grass’ in east Asian countries because they are parasitic on living insects in winter and grow out of dead underground pupae in summer. Traditionally, *Cordyceps* has been regarded as a tonic that improves energy, stamina, and libido, exhibiting a potential relationship to sex hormones and energy metabolism [24]. Among *Cordyceps* species, *Cordyceps militaris* was reported to dramatically decrease liver weight and fat deposition and improve lipid levels, suggesting that *Cordyceps militaris* can have a favorable role in regulating obesity [25]. Moreover, it was shown that *Cordyceps militaris* improves reproductive hormone concentration [26] and alleviates osteoporosis, which results from reproductive hormone deficiency [27, 28]. Therefore, *Cordyceps militaris* is expected to be able to regulate obesity induced by menopause. However, the therapeutic effect of *Cordyceps militaris* in postmenopausal obesity has not been studied thus far.

Network pharmacology is an effective method to investigate the system-level mechanism of drugs that combine multiple compounds [29]. It has been successfully used in contributing to discovering the potential mechanisms of natural products containing multiple compounds [18, 30–32]. It is expected that *Cordyceps militaris* regulates various pathways which affect the development of obesity and complications via its bioactive compounds; therefore, employing network pharmacological analysis can be effective for investigating the potential anti-obesity mechanism of *Cordyceps militaris*.

Here, we investigated whether *Cordyceps militaris* could alleviate menopause-induced obesity by combining in vivo, in vitro, and in silico methods. To explore whether *Cordyceps militaris* could relieve menopause-induced obesity, we observed the anti-obesity effects of CME in ovariectomized rats. To estimate whether *Cordyceps militaris* mitigates down-regulated estrogen receptor alpha (ERα) induced by reproductive hormone deficiency in menopause, we measured the cell proliferation and phosphorylation of ERα on MCF-7 cell lines. Finally, we investigated the underlying mechanism of *Cordyceps militaris* by analyzing the potential active compounds and pathways related to obesity and menopause.

## Methods

### Extraction of *Cordyceps militaris*

As described in our previous study [33], CME was provided by Dong-A Pharmaceutical (Yongin, Korea). Briefly, CME was extracted in a 50% ethanol (*v/v*) solution in water from *Cordyceps militaris* cultured in brown rice and concentrated under low pressure. The extract was freeze-dried for the in vitro and in vivo experiments.

### In vivo experiment

#### Animal model

Female Sprague Dawley rats ($n=64$, 7 weeks old), which were either ovariectomized or sham-operated, were provided by DooYeol Biotech (Seoul, Korea). The rats were housed in a restricted access rodent facility with up to three rats per polycarbonate cage. The rats were given seven days to acclimate before being ovariectomized (or sham-operated), and maintained on a 12-h light/dark cycle, 20–23 °C laboratory temperature, and 45–55% relative humidity. All methods were performed in accordance with the related guidelines [34] and approved by the Panel on Laboratory Animal Care of Gachon University (GIACUC-R2017037). The ARRIVE guidelines [35] were adhered to throughout this study.

#### Experimental groups and drug administration

The rats were randomly divided into the following groups: sham surgery with intact ovaries group (Sham, $n=10$); ovariectomized model group (OVX,$n=11$); 17β-estradiol-treated positive group (E2, 25 μg/kg/d,$n=10$); CME low-dose group (37.5 mg/kg/d,$n=11$); CME middle-dose group (75 mg/kg/d,$n=11$); and CME high-dose group (150 mg/kg/d, $n=11$). After one week of recovery, the four medication groups were treated with corresponding medicine, whereas the Sham and OVX groups were given an equal amount of the vehicle. The dosage was 1 ml/100 g body weight. CME was administered orally, whereas 17β-estradiol was injected intraperitoneally. Mice in each group were treated for 8 weeks. Rats were euthanized with CO_2_ gas after fasting for 12 h after eight weeks of treatment. Tissue samples were collected and kept at − 80 °C in Eppendorf tubes before assays.

#### Measurement of body weight and organ weight

The body weights of rats were measured daily during the experiment using an electronic balance (AdventurerTM, Ohaus, New Jersey, USA), in accordance with KFDA recommendations. The weights of the liver and uterus were measured using the same procedure applied to measure the body weight.

#### Serum biochemical parameters

After eight weeks of administration, blood was obtained from the abdominal veins of rats after fasting for 12 h. Blood samples were collected into CBC bottles containing EDTA-3 K (Sewon Medical, Seoul, Korea). Serum was obtained by centrifugation at 3000 × g for 15 min. High-density lipoprotein (HDL) cholesterol, low-density lipoprotein (LDL) cholesterol, total cholesterol, and triglyceride levels were measured with Accute TBA-40FR apparatus (Toshiba Medical Systems Co., Tochigi, Japan).

#### Histopathology

Histological photographs of adipose tissue were examined based on the paraffin method using a light microscope. Endometrial adipose tissue was embedded in paraffin blocks after being fixed in 10% neutral buffered formalin. Sections of 5 μm were cut and mounted on glass slides. Xylene and alcohol were used to remove the paraffin. Hematoxylin and eosin (H&E) staining were used to color the sections. After dehydration with alcohol, the photographs were obtained with a light microscope. The sizes of the endometrial adipocytes were determined with ImageJ software (Version 1.45 s, NIH, Bethesda, Maryland, USA).

#### ***Dual-energy X-ray (DXA***)

Body fat was determined in each rat by dual‐energy X‐ray absorptiometry after eight weeks of administration using an INALYZER scanner (MEDIKORS Inc., Seongnam, Gyeonggi, Korea). Rats were anesthetized by the inhalation of isoflurane during scanning.

#### Statistical analysis

Statistical significance of variation among and between groups and dose-dependency was evaluated and determined by the Kruskal–Wallis test followed by the Dunn–Bonferroni post hoc test and Jonckheere–Terpstra test using IBM SPSS Statistics (Version 25, IBM, Armonk, New York, USA), respectively. The Bonferroni correction was used to adjust for multiple comparisons. The test result was regarded as statistically significant when *p* < 0.05; *, **, *** in figures represent p-values of the test are below 0.05, 0.01, and 0.001, respectively.

### In vitro experiment

#### Cells and cell culture

The ER-positive MCF-7 human breast cancer cell line was supplied by the American Type Culture Collection (ATCC, Manassas, VA, USA). MCF-7 cells were incubated in Roswell Park Memorial Institute 1640 medium (RPMI 1640; Corning, Manassas, VA, USA), which included 10% fetal bovine serum (FBS; Gibco BRL, Carlsbad, MD, USA) and 1% penicillin/streptomycin solution (1000 IU/mL penicillin and 10,000 μg/mL streptomycin; Life Technologies, Waltham, MA, USA). Cultures were maintained at 37 °C in a humidified environment containing 5% CO_2_. Dimethyl sulfoxide (DMSO) was utilized as a solvent to dissolve the material in cell studies. In comparison to naïve cells, the final concentration of DMSO was kept below 0.1%, and there was no effect of DMSO.

#### E-screen assay

Before treatment, MCF-7 cells (1 × 10^4^ cells/well) were seeded into a 48-well plate and incubated for 24 h. Vehicle (DMSO) or indicated concentrations of *Cordyceps militaris* were dissolved in phenol red-free RPMI (Gibco, Carlsbad, CA, USA) supplemented with 10% charcoal dextran stripped serum (Innovative Research, Novi, USA). Then, the prepared samples were added to the wells and incubated for 144 h. For the antagonistic test, the ER antagonist ICI 182,780 (Tocris Bioscience, Bristol, UK) was added to the test samples. Subsequently, Ez-Cytox solution (Daeil Lab Service, Seoul, South Korea) was added to each well and the cells were cultured for 1 h. The cell viability was then calculated from the measurement of the optical density at 450 nm using a SPARK 10 M microplate reader (Tecan Group Ltd., Männedorf, Switzerland). Cell viability was calculated based on a ratio of 100% of the vehicle control (DMSO).

#### Western blot analysis

MCF-7 cells (2 × 10^5^ cells/well) were seeded into a 6-well plate and cultured for 24 h. Vehicle (DMSO) or indicated concentrations of *Cordyceps militaris* were added into the wells and cultured for 24 h. After incubation, the cells were washed with phosphate-buffered saline (PBS; Welgene, Gyeonsan, Korea) and lysed with radioimmunoprecipitation assay (RIPA) buffer (Cell Signaling Technology, Inc., MA, USA). Using a Pierce BCA Protein Assay Kit (Thermo Scientific, Carlsbad, CA, USA), the protein concentrations of lysates were measured. Equal amounts of protein were mixed with 4 × NuPAGE LDS Sample Buffer (Thermo Scientific, Carlsbad, CA, USA) and boiled for 10 min at 95 °C. The proteins were separated by precast 4–15% Mini-PROTEAN TGX (Tris–Glycine. eXtended) gel (Bio-Rad, Hercules, CA, USA) and then transferred onto polyvinylidene fluoride (PVDF) transfer membranes (Merck Millipore, Darmstadt, Germany). The membranes were blocked with 5% skim milk for 2 h. Prior to hybridization with the antibody, that membranes were cut to the corresponding site for each antibody. Each membrane was incubated with specific primary antibodies to ERα, phospho-estrogen receptor α (p-ERα), extracellular-signal-regulated kinase (ERK), phospho-ERK, p38, phospho-p38, c-Jun N-terminal kinases (JNK), and phospho-JNK, and glyceraldehyde 3-phosphate dehydrogenase (GAPDH) (Cell Signaling Technology, Inc., Danvers, MA, USA) to enhance the protein detection, followed by incubation with horseradish-peroxidase-conjugated secondary goat anti-rabbit antibody (Cell Signaling Technology, Inc., Danvers, MA, USA) for 6 h at 20 ± 5 ℃. The membranes were incubated with the horseradish secondary antibody conjugated by peroxidase (Cell Signaling Technology, Inc., Danvers, MA, USA) at 20 ± 5 ℃ for 1 h after washing. The bound antibodies were visualized with Pierce ECL Western Blotting Substrate (Rockford, IL, USA) and the FUSION Solo chemiluminescence system (PEQLAB Biotechnologie GmbH, Erlangen, Germany).

### Network pharmacology

We obtained the targets of compounds in *Cordyceps militaris* and constructed a herb–compound–target network. The herbal compound information was retrieved from TCM-MESH (http://mesh.tcm.microbioinformatics.org) [36]. We employed a method called the quantitative estimate of drug-likeness (QED), which analyzes drug-likeness based on molecular structural properties [37] to filter out compounds that are unlikely to function as drugs when administered orally. The QED scale runs from 0 to 1; a larger QED indicates more drug-likeness. A value of 0.35 is the mean QED for oral drugs approved by the Food and Drug Administration; therefore, we used it as the cut-off value for the compounds. The potential targets of the compounds were queried from STITCH (http://stitch.embl.de/). We collated the compound–protein interactions whose combined scores were over 0.7, which is considered the high-confidence criterion for filtering prediction results. Based on the herbal compound information and compound–protein interactions, we built a herb–compound–target network connecting *Cordyceps militaris* to its compounds, and the compounds to their targeting proteins. Using Enrichr and Kyoto Encyclopedia of Genes and Genomes (KEGG, https://www.genome.jp/kegg/) databases [38, 39], gene set enrichment analysis (GSEA) was conducted to investigate the relationship between targeted proteins and KEGG pathways related to estrogenic effects and obesity. We calculated the adjusted p-values and combined scores for pathways. The logarithms of the p-values and z-scores were used to calculate combined scores (notably, the combined score is different from the combined score in the STITCH).

## Results

**Fig. 1:**
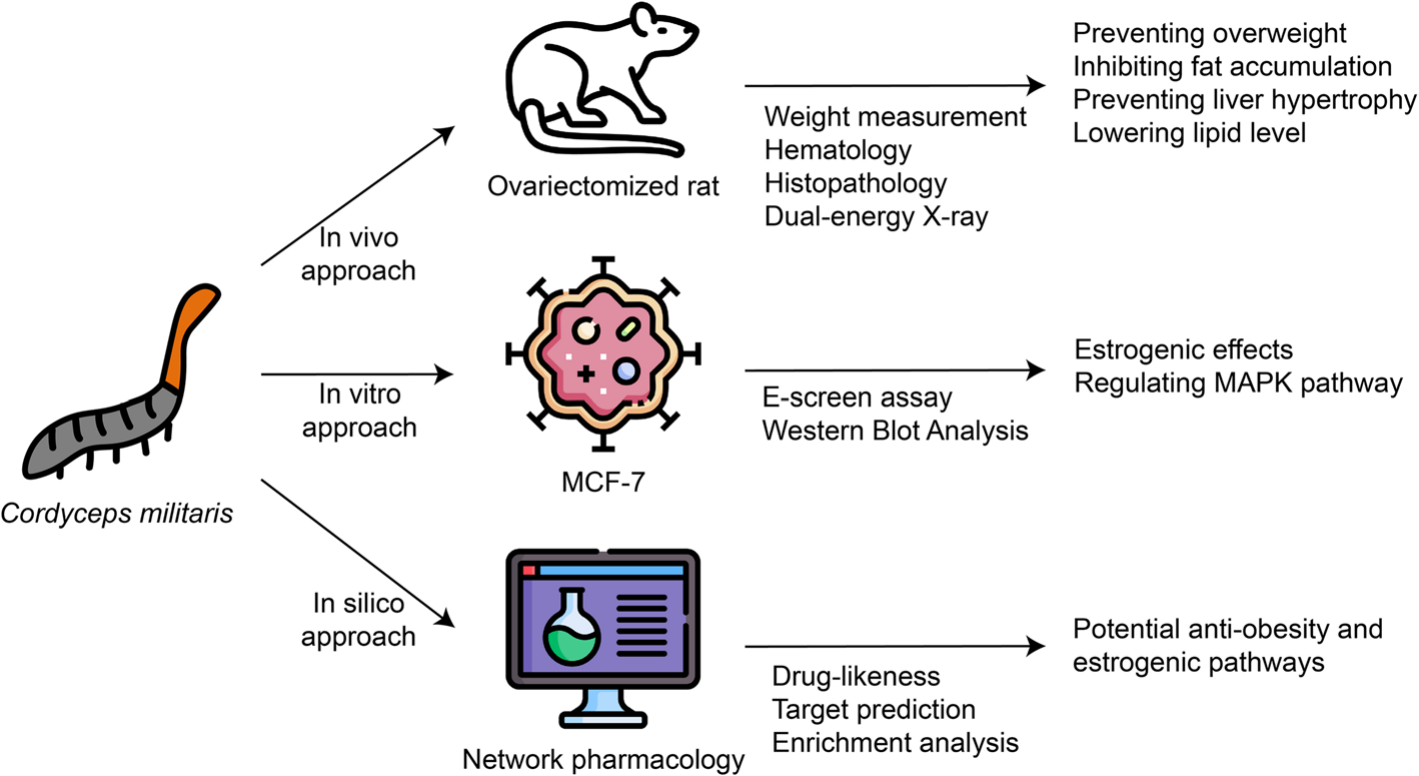
Schematic diagram to summarize the methods and results of our study

To identify the anti-obesity effects of *Cordyceps militaris* in menopause and its mechanism, we used a comprehensive method combining in vivo, in vitro, and in silico approaches (Fig. 1).

### CME showed anti-obesity effects in ovariectomized rats

**Fig. 2:**
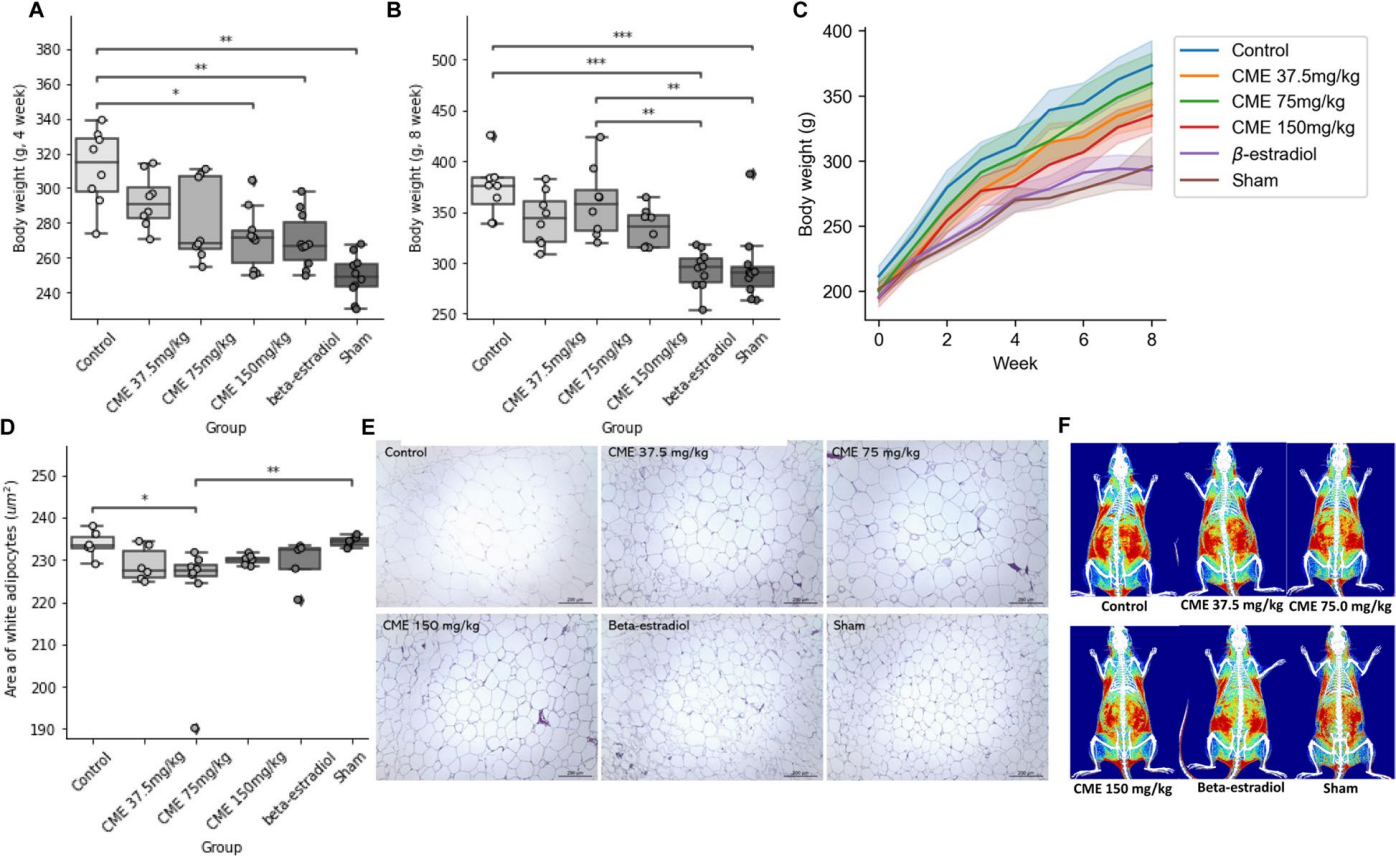
Anti-obesity effects of *Cordyceps militaris* extract (CME). **A–C** Effects of CME on body weights. Body weights after 4 weeks (**A**) and 8 weeks (**B**) are shown by the dose of CME. Dots represent individual sample weights. (**C**) Changes in body weights are represented by time. Lines and bands represent the means of weights of rats that belong to each group and their 95% confidence intervals, respectively. **D–F** Effects of CME on the accumulation of fat. (**D**) The mean area of white adipose tissue after 8 weeks of administration is shown by the dose of CME. All other details are the same as in (A). (**E**) Representative results of white adipose tissue histology. (**F**) Representative results of dual-energy X-ray. The red-colored region represents where fat is highly accumulated. *p < 0.05, **p < 0.01, ***p < 0.001 between the two groups

Ovariectomized rats were treated with CME for eight weeks, and their body weights were measured daily. As a result, body weights in the high-dose CME group and positive controls were shown to be significantly lowered, and the high-dose CME group and positive controls showed similar body weight loss effects at week 4 (Fig. 2A). Meanwhile, no significant differences were found between the body weights of CME groups and controls at week 8 in the Dunn–Bonferroni post hoc tests followed by Kruskal–Wallis tests (Fig. 2B). However, CME exhibited weight loss effects in a dose-dependent manner at week 4 (*p* = 0.014) and week 8 (*p* = 0.024) in Jonckheere–Terpstra tests. This trend continued during the whole experimental period (Fig. 2C), suggesting that CME prevented overweight in ovariectomized rats. We also measured the area of white adipocytes. The area of white adipocytes in the middle-dose CME group was shown to be lowered compared with the control and sham groups (Fig. 2D). However, the hypertrophy of white adipocytes was not induced by ovariectomy, and although the area of white adipocytes decreased, it was not statistically significant (*p* = 0.209). The difference in the area of white adipocytes between groups is representatively shown in Fig. 2E. In Fig. 2F, it is representatively shown that rats in middle-dose and high-dose CME groups had less fat accumulation than those in the control group.

**Fig. 3:**
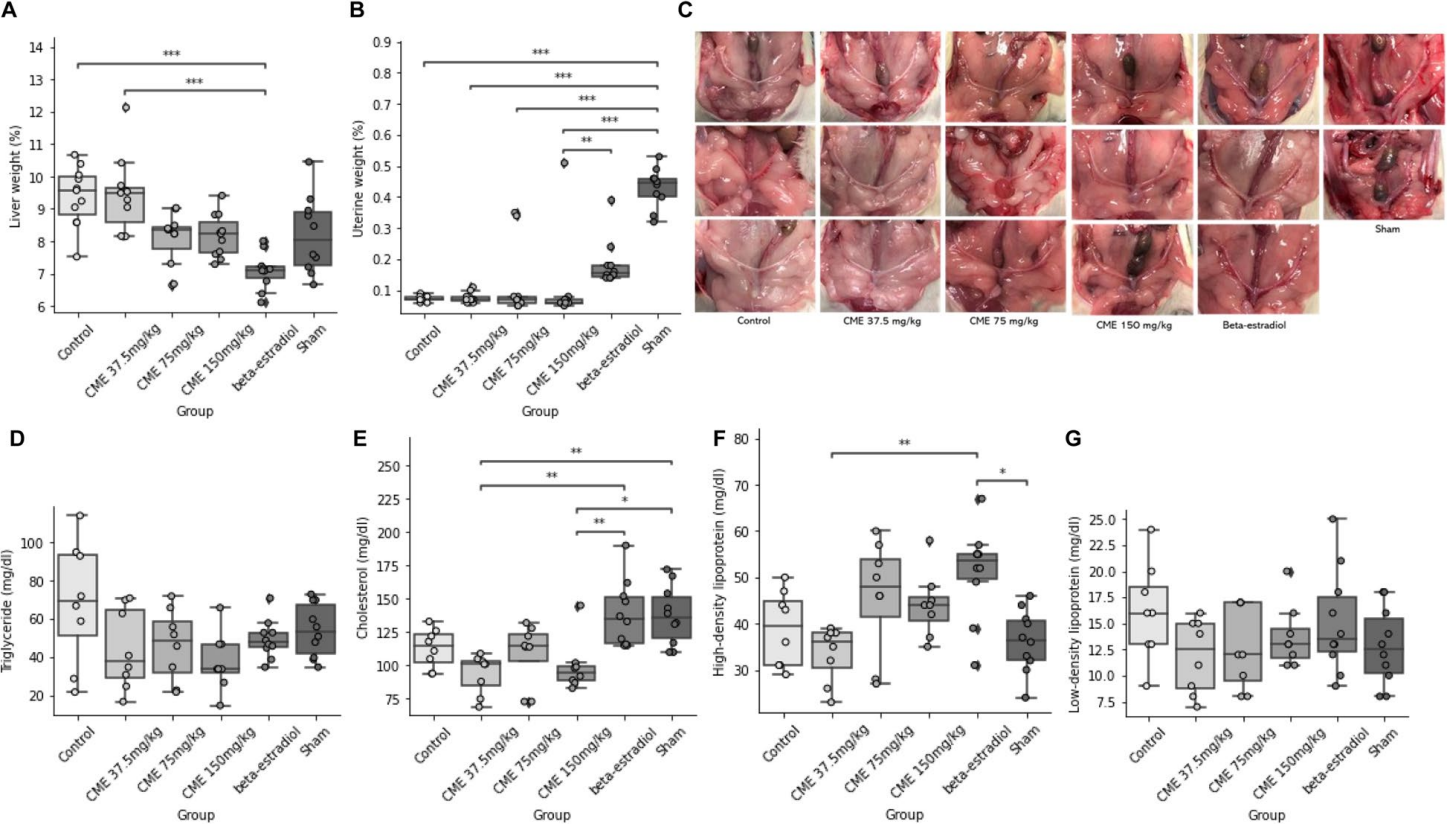
Effects of *Cordyceps militaris* extract (CME) on the weights of organs and lipid levels. **A–C** Effects of CME on the weights of organs. Weights of the liver (**A**) and uterus (**B**) after 8 weeks of administration are shown by the dose of CME. All other details are the same as in Fig. 2. (**C**) Representative results of uterine morphology after 8 weeks of administration. **D–G** Effects of CME on lipid levels. Levels of triglyceride (**D**), cholesterol (**E**), high-density lipoprotein (**F**), and low-density lipoprotein (**G**) after 8 weeks of administration are shown by the dose of CME. All other details are the same as in Fig. 2. *p < 0.05, **p < 0.01, ***p < 0.001 between the two groups

We also measured the liver and uterine weights. Liver weights in each CME group were shown to be non-significantly lower compared with the controls; however, liver weights in the CME groups were lowered in a dose-dependent manner (*p* < 0.001). Notably, the liver weights of the middle-dose and high-dose CME groups were close to those of the sham group (Fig. 3A). This suggests that CME prevents liver hypertrophy, which was observed in OVX rats. The uterus weight rates in the CME groups did not increase compared with those in sham and positive control groups, whereas the uterus weights of the beta-estradiol group showed a trend of increase (Figs. 3B and 3C). This suggests that CME does not act as an ER agonist for the growth of the uterus. Additionally, we measured lipid levels and found that levels of triglyceride in CME groups were significantly decreased in a dose-dependent manner (*p* = 0.047), although each CME group showed no significant difference compared with the control (Fig. 3D). For other lipid levels including cholesterol, HDL, and LDL, CME showed neither significant differences compared with controls nor improvements in lipid levels in a dose-dependent manner (Fig. 3E-G). Taken together, our results propose that CME improves liver hypertrophy and triglyceride levels in a dose-dependent manner.

### CME interacts with ERα and MAPK in MCF-7

**Fig. 4:**
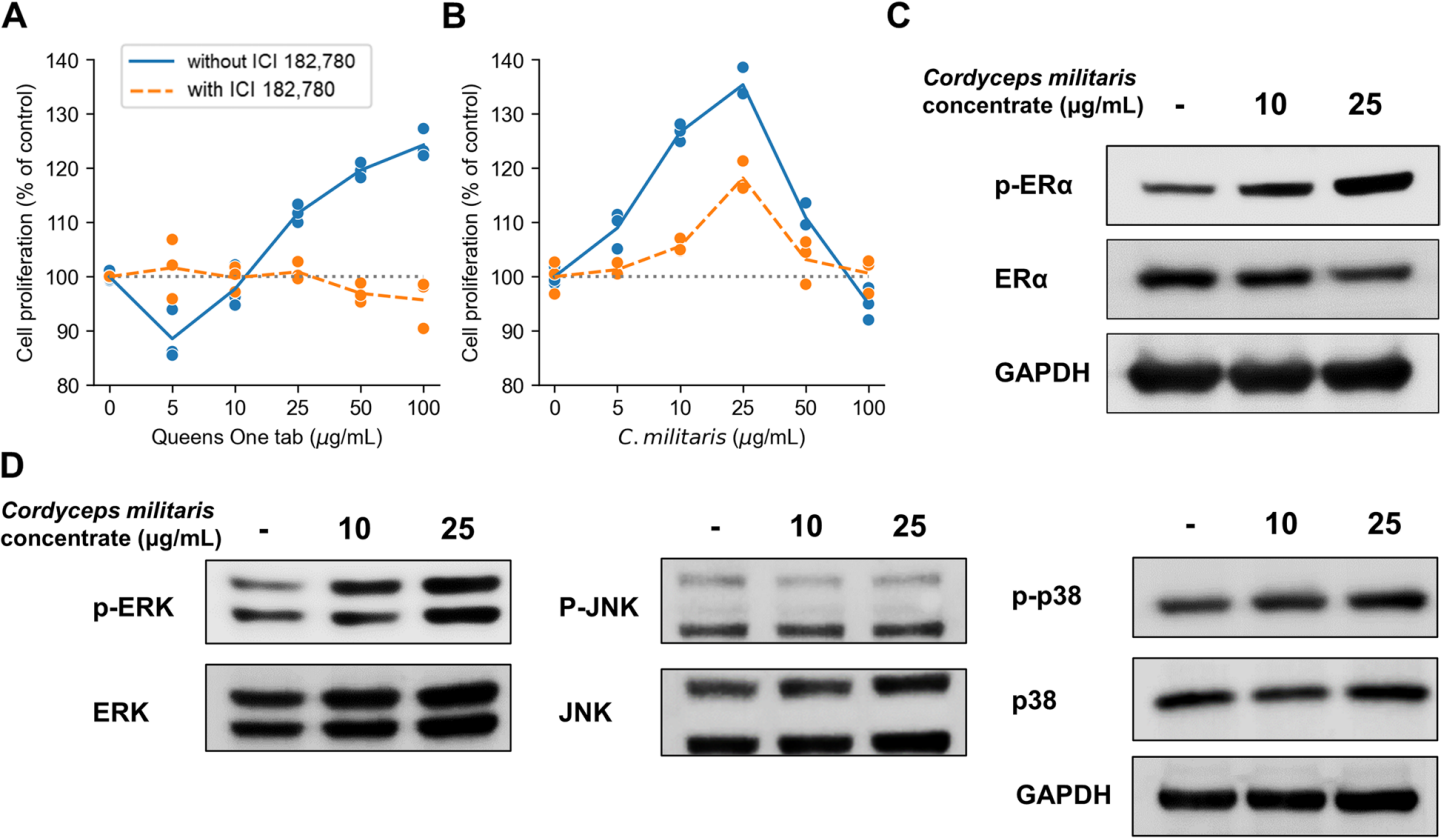
Effect of *Cordyceps militaris* extract (CME) on proliferation in MCF-7 cells. Relative cell proliferation ratios are shown by the dose of (**A**) Queens One tab, used as a positive control, and (**B**) CME for 24 h. All other details are the same as in Fig. 2. **C** Levels of estrogen receptor α (ERα) phosphorylation in MCF-7 cells, determined by Western blotting, are shown by the dose of CME. Levels of protein expression of phospho-estrogen receptor α (p-ERα) are compared with levels of ERα and glyceraldehyde 3-phosphate dehydrogenase (GAPDH). **D** Levels of phosphorylation of mitogen-activated protein kinases in MCF-7 cells, determined by Western blotting, are shown by the dose of CME. Levels of protein expression of phospho-extracellular-signal-regulated kinase (ERK), p- c-Jun N-terminal kinases (JNK), p-p38 are compared with levels of Total ERK, JNK, p38. Uncropped images are shown in Supplementary Figure S1

Next, we investigated the possibility of whether CME can mitigate the lowered estrogenic activity. We measured the estrogenic activity of CME on an ER-positive MCF-7 human breast cancer cell line through a modified Soto’s E-screen assay [40]. The MCF-7 cells were exposed to 10 and 25 μg/mL of CME for 144 h. Queens One tab. (positive control), which is an extract of *Trifolium pratense* that exhibits estrogenic effects in vivo in ovariectomized Sprague Dawley rats [41], increased the MCF-7 cell proliferation in a concentration-dependent manner under 25 μg/mL (Fig. 4A). The effect was completely inhibited by co-treatment with ICI 182,780, which is an ER antagonist. Therefore, the cell proliferation induced by the Queens One tab. is mediated by ERs. Similarly, the CME was also shown to increase cell proliferation in a dose-dependent manner (Fig. 4B), although the effect was limited to doses of less than 25 µg/mL. The effect was also attenuated by co-treatment with ICI 182,780, but not completely inhibited. These results imply that CME acts as a partial estrogenic agent.

ERα, also called estrogen receptor 1 (ESR1), is a ligand-dependent nuclear hormone receptor transcription factor. When ERα is bound with 17β-estradiol (E_2_), a ligand of ERα, ERα binds to specific DNA sequences called estrogen response elements with high affinity. [42]. Generally, ligands of ERα, such as E_2_, induce the phosphorylation of Serine 118 in ERα [43]. Thus, we performed Western blotting to investigate the activation of ERα by CME. As shown in Fig. 4C, CME increased the expression of ERα phosphorylation. The results indicate that the treatment of CME induces phosphorylation with serine residues of ERα. Also, CME increased the expression of EKR and p38 phosphorylation among MAPKs (Fig. 4D). The results indicate that the treatment of CME induces phosphorylation with ERK and p38.

### Compounds in *Cordyceps militaris* are associated with multiple pathways

**Fig. 5:**
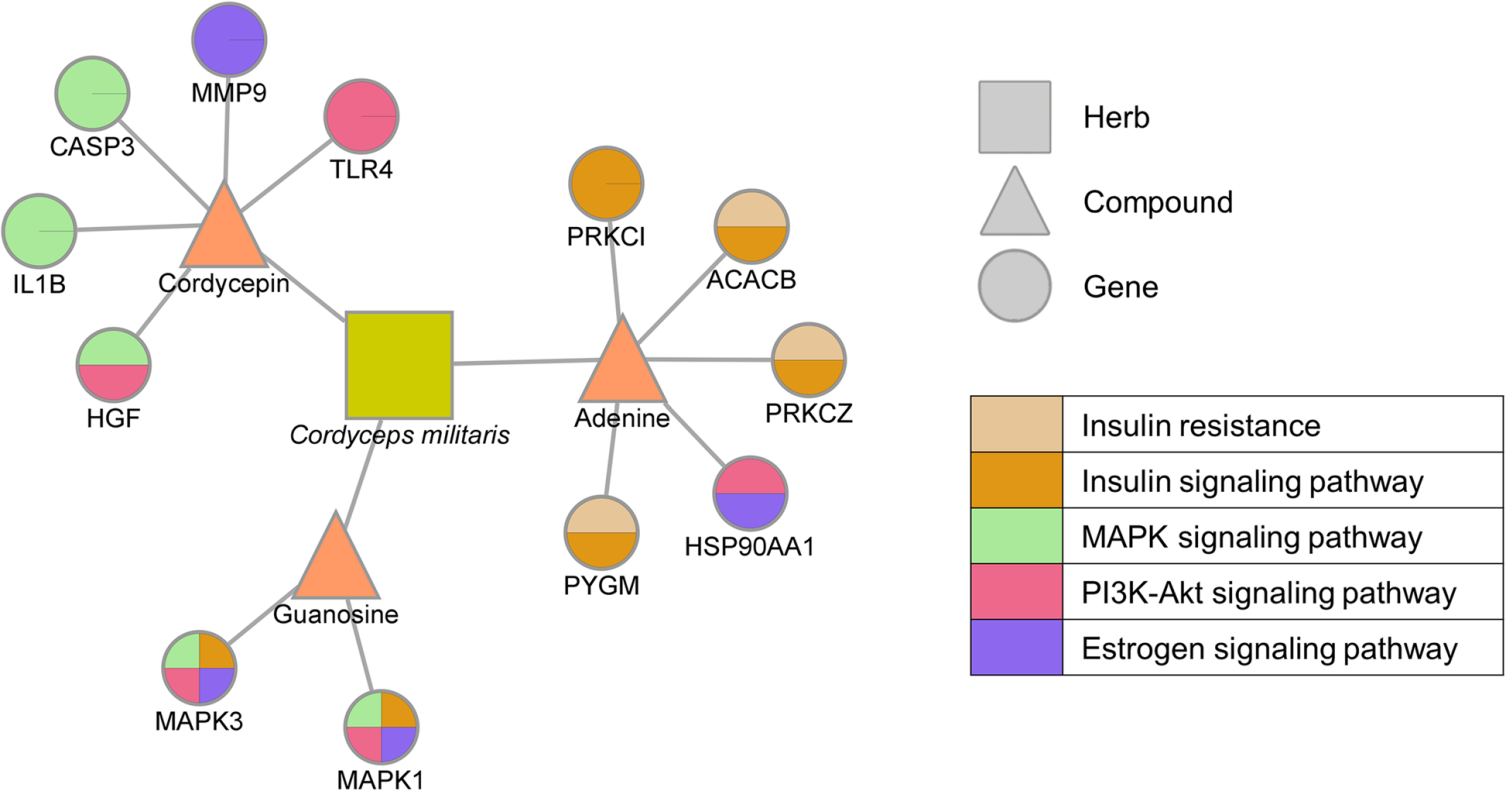
Herb–compound–target network of *Cordyceps militaris*. Edges between the herbs and compounds represent the herbs that contain the compounds. Edges between the compounds and genes represent genes that are the predicted targets of the compounds. Genes are colored by their related pathways. Note that we only visualized compounds and genes related to the potential pathways

**Fig. 6:**
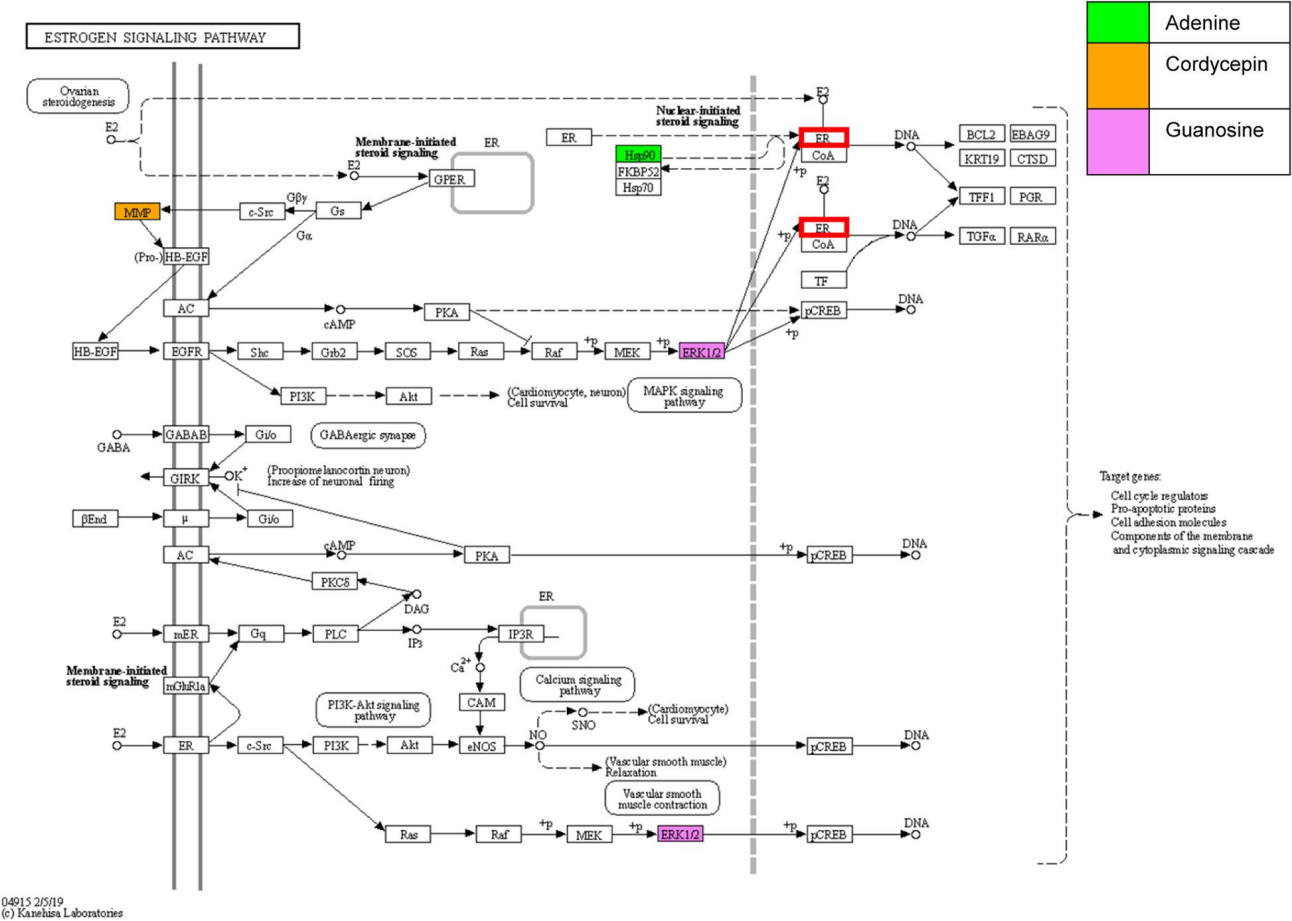
Estrogen signaling pathway and the targets of compounds contained in *Cordyceps militaris*. Colored genes represent the predicted targets of compounds in *Cordyceps militaris*. Red border boxes represent estrogen receptor α, whose phosphorylation is described above

Finally, we investigated the potential pathway of CME which alleviates obesity and related symptoms induced by menopause by employing a network pharmacological approach. We constructed a herb–compound–target network which consisted of 117 nodes and 152 edges. The nodes correspond to the herb, potential bioactive compounds, or their targets. Additionally, the edges indicated the inclusiveness of compounds (between herbs and compounds) or interactions (between compounds and targets). We conducted enrichment analyses of the KEGG pathways related to obesity or estrogen with the predicted targets of compounds of *Cordyceps militaris*. Our results showed that these predicted targets are associated with multiple pathways, such as the insulin signaling pathway, phosphatidylinositol 3 kinase (PI3K)–Akt signaling pathway, the mitogen-activated protein kinase (MAPK) signaling pathway, the estrogen signaling pathway, and insulin resistance, which are related to obesity and the menopause (Table 1). This association indicates that *Cordyceps militaris* could alleviate obesity and obesity-related symptoms via regulating these pathways. Additionally, compounds in *Cordyceps militaris* were predicted to interact with mutually exclusive groups of genes related to obesity. Adenine was predicted to interact with protein kinase C iota (PRKCI), acetyl-CoA carboxylase beta (ACACB), protein kinase C zeta (PRKCZ), glycogen phosphorylase, muscle-associated (PYGM), and heat shock protein 90 alpha family class A member 1 (HSP90AA1), which are mainly associated with insulin-related pathways. Meanwhile, cordycepin was predicted to interact with caspase 3 (CASP3), interleukin 1 beta (IL1B), hepatocyte growth factor (HGF), matrix metallopeptidase 9 (MMP9), and Toll-like receptor 4 (TLR4), which are associated with signaling pathways of MAPK, PI3K–Akt, and estrogen. Guanosine was predicted to interact with MAPK3 and MAPK1, which are associated with various biological pathways. We note that predicted targets of compounds that are related to obesity in menopause are mutually exclusive (Fig. 5). In the case of the estrogen signaling pathway, the compounds were predicted to interact with mutually exclusive targets, which can affect ERs (Fig. 6). These results suggest that compounds in *Cordyceps militaris* could regulate estrogen-related pathways in a complementary manner.

**Table 1:** Enrichment analysis of KEGG pathways related to obesity or estrogen through the targets of compounds of *Cordyceps militaris*

Term	Overlap	Rank	*P*-value (adjusted)	Odd ratio	Combined Score	Genes
Insulin signaling pathway	6/137	28/192	0.0007	8.87	81.73	PRKCI; MAPK1; PYGM; PRKCZ; ACACB; MAPK3
PI3K–Akt signaling pathway	7/354	54/192	0.0109	3.90	22.54	CASP9; HSP90AA1; BDNF; HGF; MAPK1; TLR4; MAPK3
MAPK signaling pathway	6/295	60/192	0.0168	3.99	20.88	BDNF; HGF; IL1B; CASP3; MAPK1; MAPK3
Estrogen signaling pathway	4/137	65/192	0.0191	5.71	28.76	HSP90AA1; MAPK1; MMP9; MAPK3
Insulin resistance	3/108	89/192	0.0445	5.38	20.86	PYGM; PRKCZ; ACACB

## Discussion

Compounds in *Cordyceps militaris* have been determined by several methods, including multi-column liquid chromatography, linear ion trap liquid chromatography-tandem mass spectrometry, and ion-pairing reversed-phase liquid chromatography-mass spectrometry [44–46]. It has been reported that adenosine monophosphate (AMP), phenylalanine, uridine, hypoxanthine, inosine, guanine, guanosine, dAMP, adenosine, adenine, cordycepin, cytosine, cytidine, uracil, hypoxanthine, 2′-deoxyguanosine, inosine, 2′-deoxyuridine, β-thymidine were detected. Among them, cordycepin is mostly considered a key component of *Cordyceps* species, including *Cordyceps militaris* [47–50]. Cordycepin binds to a number of intracellular targets, including DNA/RNA, influencing apoptosis and the cell cycle. Cordycepin typically exerts therapeutic effects on tumors by inhibiting the DNA-binding activity of activator protein-1 and NF-κB [51]  or alleviating inflammation by reducing the production of inflammatory mediators such as nitric oxide, prostaglandin E2, TNF-α, and IL-1β, and downregulating iNOS, COX-2, and TNF-α gene expression [51, 52]. The roles of guanosine and adenine in the therapeutic mechanisms of *Cordyceps* species are less well known. However, in the case of guanosine, it has been found to exert neuroprotective [53] and anti-inflammatory properties [54, 55], implying that it may be a potential therapeutic compound for *Cordyceps* species.

Hormonal changes during the menopausal period lead to various symptoms, including obesity [1, 3, 56]. Obesity not only worsens the quality of life but also constitutes a risk factor for metabolic and cardiovascular diseases [56]. Moreover, adipocytokines synthesized in adipocytes are considered to be modulators of insulin resistance and chronic inflammation [57]. Therefore, regulating obesity after menopause is an important issue in women’s health. In our study, we investigated whether *Cordyceps militaris* can regulate obesity induced by menopause. We found that CME prevented obesity and improved some lipid levels dose-dependently in ovariectomized rats. CME also exhibited ER agonistic effects in in vitro experiments. Through network pharmacological analysis, it was shown that the bioactive compounds in *Cordyceps militaris* are associated with multiple pathways which can affect obesity and its complications. Our results suggest that CME could be used for treating postmenopausal obesity in women.

Concerning white adipocytes, ovariectomies are generally believed to induce obesity, inducing the hypertrophy of white adipocytes. However, regardless of the induction of obesity, the hypertrophy of white adipocytes may not be triggered within certain experimental settings, such as postoperative terms [58]. Our results indicate that ovariectomized rats had significant increases in body weight, but no increases in white adipocyte size. Therefore, additional research is required to ascertain the relationship between ovariectomy and white adipocytes, as well as the relationship between CME and white adipocytes.

Although controversial, obesity in menopause is considered to result from a deficiency of estrogen. Indeed, estrogen-based HRT in menopausal women has been reported to beneficially affect lipid levels, fasting serum glucose, insulin levels, and abdominal fat [4, 12, 59, 60]. It is well known that the ventrolateral portion of the ventral medial nucleus (VL VMN) and the arcuate in the hypothalamus control energy intake and expenditure, and a decrease in ERα in VL VMN coincides with an increase in adiposity and loss in energy expenditure [61]. Moreover, it has been reported that estrogen can regulate food intake in the hindbrain [62]. Our study showed that CME significantly increased the phosphorylation of ERα at doses of 10 µg/ml and 25 µg/ml in the MCF-7 cell, suggesting that the anti-obesity effects of CME may be based on its ERα agonistic activity. Interestingly, CME did not exert estrogenic effects on uterine development in ovariectomized rats in this study. This suggests that CME could selectively activate ERα depending on the types of tissues. There is a possibility that CME regulates food intake and energy expenditure by activating ER in the central nervous system, while not activating those in uterus. Additionally, the result that CME fostered the proliferation of MCF-7 indicates that caution should be exercised when applying CME therapy for ER-positive breast cancer. Selective action of CME is a key area to be explored further. Additionally, the network pharmacological method predicted that cordycepin would interact with matrix metallopeptidase 9 (MMP9), whose pathway is related to MAPK and ERα. Moreover, adenine and guanosine, which are the other predicted bioactive compounds of *Cordyceps militaris*, were predicted to be related to HSP90A1 and MAPK, respectively. The MAPK and estrogen signaling pathways are closely related. It has been demonstrated that the expression of MAPK increased ERα-induced transcriptional activation, suggesting that MAPK/ER cross-talk enhances the estrogen signaling pathway [63]. Therefore, these results imply the possibility that the compounds in CME may synergistically enhance estrogenic pathways through MAPK regulation. However, the diverse experimental settings and the limited scope of studies that explore the relationship between *Cordyceps* and estrogen have often provided conflicting evidence. For example, one study reported that *Cordyceps militaris* had antimetastatic effects by inhibiting estrogen-related receptor alpha (ERRα) [64]; on the other hand, another study reported that *Cordyceps sinensis* alleviated osteoporosis induced by estrogen deficiency [65]. Some studies have reported that cordycepin, one of the predicted bioactive compounds of *Cordyceps militaris*, inhibits the growth of MCF-7 and the phosphorylation of ERRα in ovarian carcinoma cells [64, 66]. Therefore, future studies should focus on the relationship between the bioactive compounds of *Cordyceps militaris* and the estrogen signaling pathway.

Obesity is related to the risk of developing insulin resistance and type 2 diabetes [67]. Adipose tissue in obesity releases higher amounts of non-esterified fatty acid, glycerol, hormones, and adipocytokines involved in the development of insulin resistance [68, 69]. Obesity, type 2 diabetes, and cardiovascular diseases share a common metabolic environment characterized by insulin resistance [68]; therefore, regulating insulin resistance and secretion could be a key mechanism to prevent the aggravation of obesity and its related metabolic diseases. Cordyceps have been reported to improve insulin resistance, insulin secretion, and glucose level [70–72]. In our prediction, adenine regulates insulin-related genes such as acetyl-CoA carboxylase β and glycogen phosphorylase B, whereas guanosine and cordycepin regulate the MAPK and PI3Ks-Akt signaling pathways. MAPK, PI3Ks, and Akt are known to be associated with obesity and its complications, including type 2 diabetes and non-alcoholic fatty livers [73–77]. These results imply that *Cordyceps militaris* may alleviate obesity and its complications through its compounds which synergistically regulate the signaling pathways of estrogen, insulin, MAPK, and PI3K–Akt.

## Conclusions

Our study investigated the therapeutic effect of CME on obesity and its mechanisms. CME alleviated obesity and its related symptoms in ovariectomized rats. CME exhibited estrogen agonistic activity in MCF-7 cell lines. Additionally, we predicted the bioactive compounds and their potential pathways to obesity. This study contributes to developing the understanding of the anti-obesity effects and mechanism of *Cordyceps militaris*. We also suggest that our approach combining in vivo, in vitro, and in silico methods is a reliable strategy to explore the efficacy and mechanisms of herbal medicine.

## Supplementary Information

**Additional file 1. Supplementary Figure S1.** Levels of estrogen receptor α (ERα) and MAPKs phosphorylation in MCF-7 cells, determined by western blotting, are shown by the dose of CME.

## Data Availability

The datasets used and/or analyzed during the current study are available from the corresponding author upon reasonable request.

## Abbreviations

HRT: Hormone Replacement Therapy
ER: Estrogen Receptor
CME: *Cordyceps militaris* Extract
OVX: Ovariectomized Model Group
ERα: Estrogen Receptor Alpha
HDL: High-Density Lipoprotein
LDL: Low-Density Lipoprotein
H&E: Hematoxylin and Eosin
DXA: Dual-energy X-ray
SD: Standard Deviation
DMSO: Dimethyl Sulfoxide
RIPA: Radioimmunoprecipitation Assay
PVDF: Polyvinylidene Fluoride
p-ERα: Phospho-Estrogen Receptor α
ERK: Extracellular-signal-regulated kinase
JNK: Jun N-terminal Kinase
GAPDH: Glyceraldehyde 3-Phosphate Dehydrogenase
QED: Quantitative Estimate of Drug-likeness
GSEA: Gene Set Enrichment Analysis
ESR1: Estrogen Receptor 1
E_2_ 17β: Estradiol
MAPK: Mitogen-Activated Protein Kinase
PRKCI: Protein Kinase C Iota
ACACB: Acetyl-CoA Carboxylase Beta
PRKCZ: Protein Kinase C Zeta
PYGM: Glycogen Phosphorylase, Muscle Associated
HSP90AA1: Heat Shock Protein 90 Alpha Family Class A Member 1
CASP3: Caspase 3
IL1B: Interleukin 1 Beta
HGF: Hepatocyte Growth Factor
MMP9: Matrix Metallopeptidase 9
TLR4: Toll-Like Receptor 4
VL VMN: Ventrolateral Portion of the Ventral Medial Nucleus
ERRα: Estrogen-Related Receptor Alpha
