# Supplementary material for: System-level investigation of anti-obesity effects and the potential pathways of Cordyceps militaris in ovariectomized rats
Source: BMC Complement Med Ther. 2022 May 12;22:132. doi: 10.1186/s12906-022-03608-y (PMC9102749; doi:10.1186/s12906-022-03608-y)
Supplement: Supplementary file 1 — Additional file 1. Supplementary Figure S1. Levels of estrogen receptor α (ERα) and MAPKs phosphorylation in MCF-7 cells, determined by western blotting, are shown by the dose of CME. [file 12906_2022_3608_MOESM1_ESM.docx]

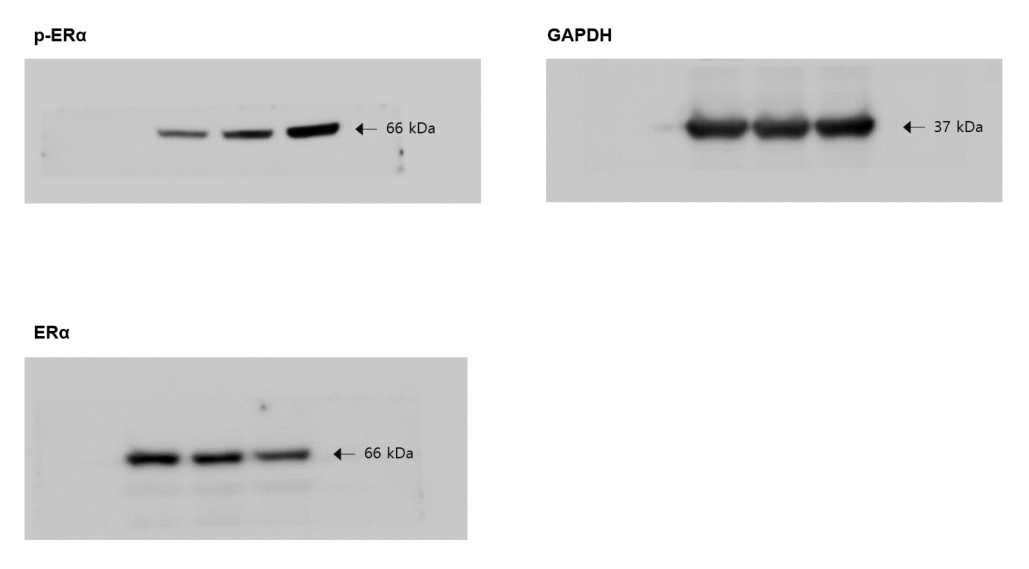


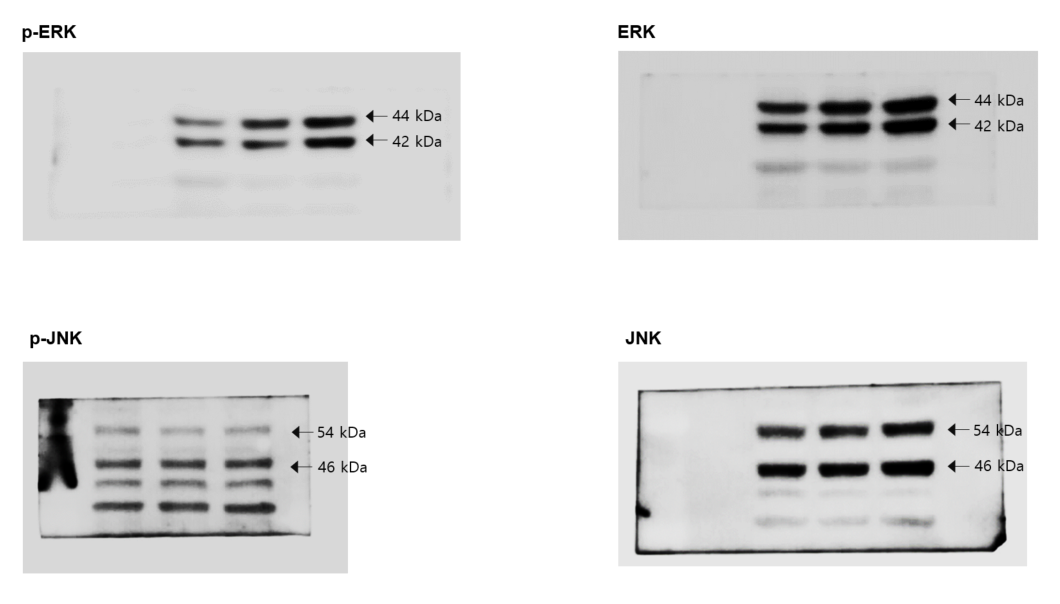


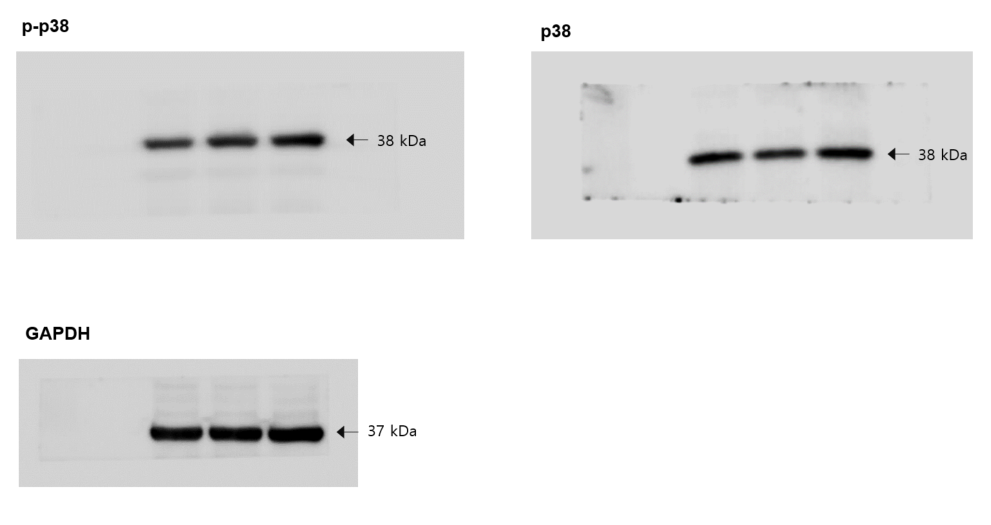


Supplementary Figure S1. Levels of estrogen receptor α (ERα) and MAPKs phosphorylation in MCF-7 cells, determined by western blotting, are shown by the dose of CME.
